# Functioning and Disability Profile of Children with Microcephaly Associated with Congenital Zika Virus Infection

**DOI:** 10.3390/ijerph15061107

**Published:** 2018-05-29

**Authors:** Haryelle Náryma Confessor Ferreira, Veronica Schiariti, Isabelly Cristina Rodrigues Regalado, Klayton Galante Sousa, Silvana Alves Pereira, Carla Patrícia Novaes dos Santos Fechine, Egmar Longo

**Affiliations:** 1 Postgraduate Program in Collective Health, Federal University of Rio Grande do Norte—Faculty of Health Sciences of Trairi (UFRN-FACISA), Santa Cruz 59200-000, Brazil; haryelle_naryma@yahoo.com.br (H.N.C.F.); galante@facisa.ufrn.br (K.G.S.); apsilvana@gmail.com (S.A.P.); 2 Division of Medical Sciences, University of Victoria, BC V8W 2Y2, Canada; vschiariti@cw.bc.ca; 3 Postgraduate Program in Physical Therapy, Federal University of Rio Grande do Norte, Natal 59078-970, Brazil; isabellyrodrigues_@hotmail.com; 4 Undegraduate Program in Physical Therapy, UNIPÊ, João Pessoa 58053-000, Brazil; carla.novaes@unipe.br

**Keywords:** ICF, public health, Zika virus infection, microcephaly, children

## Abstract

Introduction: The increase in the number of cases of microcephaly in Brazil and its association with the Zika virus (ZIKV) is a global public health problem. The International Classification of Functioning Disability and Health (ICF) model is a powerful tool and extremely relevant in managing disability. Objective: Describe the functioning profile of children with microcephaly associated with ZIKV in two states of northeastern Brazil. Methods: This is a descriptive cross-sectional study. The sociodemographic characteristics, head circumference, and other clinical data were collected from medical charts, physical examinations, measuring instruments, and interviews with the children and their parents. The Brazilian Portuguese version of the Brief Common ICF Core Set for cerebral palsy (CP) was used. Each ICF category was assigned a qualifier, which ranged from 0 to 4 (no problem, mild problem, moderate problem, severe problem, complete problem). For environmental factors, 0 represents no barrier and 4 represents complete barrier; +0, no facilitator and +4, complete facilitator. Results: A total of 34 children with microcephaly caused by ZIKV were recruited (18 girls and 16 boys) at four rehabilitation facilities in Rio Grande do Norte and Paraíba states, Brazil. The average age of the participants was 21 months, monthly income was ≈USD 300.00, and head circumference z-scores ranged between 0.92 and −5.51. The functioning profile revealed complete disability in most of the body function categories (b). The activity and participation areas (d) were highly impacted, particularly in mobility-related categories. With respect to environmental factors (e), most of the sample reported a complete facilitator for the immediate family, friends, and health services, systems, and policies, as well as a complete barrier to societal attitudes. Conclusion: This is the first study that describes the functioning profile of children with microcephaly associated with ZIKV, using a tool based on the ICF in Brazil. Our findings reinforce the need to maximize health care and access to information, based on the ICF, for multiprofessional teams, administrators, family members, and children.

## 1. Introduction

In late 2015, public health worldwide focused its attention on Brazil due to an outbreak of the Zika Virus (ZIKV) that caused an increase in cases of microcephaly in the northeastern region of the country, which led the Brazilian Ministry of Health to declare a public health emergency [1]. In January 2016, 3530 suspected cases of microcephaly were notified, many of which occurred in children born of women who lived in or visited ZIKV transmission areas [2]. According to epidemiological bulletin no. 29–Epidemiological Week, no. 22/2016, 113 and 136 confirmed cases of microcephaly after ZIKV infection were reported by the Ministry of Health in the states of Rio Grande do Norte and Paraíba, respectively [3].

The Ministry of Health has developed numerous strategies to combat ZIKV, aimed at providing care to children with microcephaly and their families [2]. With respect to health care, in addition to health education initiatives, the Ministry of Health broadened sexual and reproductive measures by offering free contraceptives, conducting home visits to educate the population, and monitoring women during pregnancy and the postpartum period. Control measures were also implemented, including eliminating possible mosquito breeding grounds, cleaning vacant lots, discarding trash, and the proper use of water-bearing containers [4].

Child development depends on both biology and the environment the children are brought up in. The likelihood of developmental problems occurring increases between 90% and 100% when a child is exposed to a series of 6–7 risk factors including poverty, mental disease in the caregiver, abuse, single parenting, and low maternal schooling [5]. In fact, there is strong evidence for the association between disability and poverty in low- and middle-income countries, which demands an urgent need for policies and programmatic actions to break this cycle [6,7]. In this respect, the Ministry of Health recommends that, in addition to parental monitoring, children with microcephaly after ZIKV infection should also be referred for early stimulation at rehabilitation centers (Specialized Rehabilitation Center, Center for Rehabilitation in Physical Medicine—intermediate level, Intellectual Rehabilitation Service) by pediatric physical therapy, speech therapists, or occupational therapists from Basic Care teams or at the Outpatient Facility for the Follow-up of At-risk Newborns [4].

There is still considerable uncertainty about the long-term neurological results and developmental trajectories of children congenically infected with ZIKV [5]. Microcephaly, in turn, does not have a very specific diagnosis, and all reported cases are considered suspect until confirmation by neurological diagnosis using computerized tomography or magnetic resonance. However, understanding and dealing with such an unexpected, extreme, and rapidly expanding phenomenon requires the joint efforts of national and international scientific communities, health policy formulators, and funding entities [8].

The International Classification of Functioning Disability and Health (ICF) model has emerged as a powerful tool that is extremely important in managing functioning and disability. The ICF was published by the World Health Organization (WHO) in 2001 [9] and has been an important instrument in classifying health conditions and promoting social inclusion policies. Its use was recommended since it is a reference framework that best reflects the principles and values of the biopsychosocial model, understanding functioning and disability as a dynamic interaction between health problems and contextual factors, both personal and environmental [9,10,11].

A number of strategies have been proposed to incorporate the ICF into public health and rehabilitation services. ICF-based tools, like the ICF core sets, provide a group of categories relevant to specific health conditions for an effective and objective assessment of functioning and disability. The ICF core sets for cerebral palsy (CP), for example, were developed by Schiariti et al., in 2014 [12], originally in English, and subsequently translated into several languages, including Brazilian Portuguese.

Trabacca et al. (2012) [13] underscores that the use of ICF core sets operationalizes clinical practice and research, making them more time-efficient. Furthermore, incorporating the influence of environmental and personal factors on functional skills results in endless potential for therapeutic interventions: for example, behavioral motivation interventions and the early introduction of assistive technology [14].

Considering the growing number of children at rehabilitation services with ZIKV-related microcephaly, whose clinical characteristics include spasticity and other signs of pyramidal liberation consistent with CP [2,15,16], the abbreviated ICF core set for CP emerges as a valid framework to guide the developmental assessment of this population. As such, the present study aims to describe the functioning and disability profile of children with microcephaly associated with ZIKV using the Common Brief ICF Core Set for CP, in two states in northeastern Brazil.

## 2. Methods

### 2.1. Participants and Procedures

**Figure 1:**
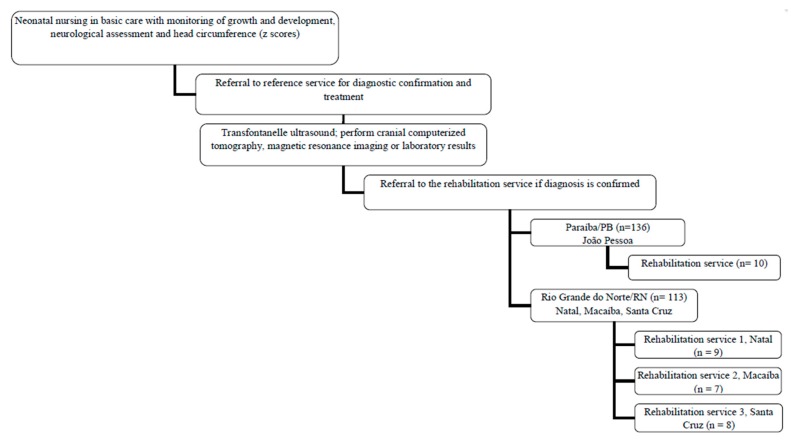
Flowchart of confirmed cases of newborn with microcephaly suggestive of being related to congenital infection by Zika Virus (ZIKV)

This is a descriptive cross-sectional study. The convenience sample consisted of 34 children with ZIKV-associated microcephaly in two states of northeastern Brazil, treated at four rehabilitation services in Paraíba (1) and Rio Grande do Norte states (3). The secondary care rehabilitation centers located in the cities of Natal, Macaíba and Santa Cruz (Rio Grande do Norte); and João Pessoa (Paraíba) were invited to participate in the study as treatment centers for children notified by the Ministry of Health and referred by surveillance service of the municipality. The flowchart of notification, diagnostic confirmation, and referral to rehabilitation centers is illustrated in Figure 1.

Each participating center held an ICF and Common Brief ICF core set for CP [12] training workshop based on the ICF educational e-tool (http://learn.phsa.ca/shhc/icf/story_html5.html) developed by Schiariti et al., 2015 [17]. Training was conducted by E.L and H.F. (first and last authors) in a 16-h module aimed at the health professionals involved in the study, as an effort to consolidate the theoretical and practical concepts on the ICF, aiming to ensure high inter-rater reliability of the qualifiers in each ICF core set category. Data collection occurred between September 2017 and January 2018, with an average duration of one hour.

### 2.2. Assessment Measures

The Portuguese version of the Common Brief ICF Core Set for CP was used (Complete list of categories and definitions is included in Figure S1). The instrument contains 25 categories: eight related to body functions (b), one to body structure (s), eight to activity and participation (d), and eight to environmental factors (e). Each ICF category received a qualifier ranging from 0 to 4 (no problem, mild problem, moderate problem, severe problem, complete problem). In addition, the qualifier 9 was assigned when information was not applicable, for example due to the age of the child the developmental milestone was not expected to have been reached, as follows; d450 Walking and d530 Toileting. For the d450 category, the qualifier 9 was used for children aged 20 months or less, while for the d530 category, 9 was considered for age equal to or greater than 24 months (arbitrary cut off). For environmental factors, 0 represents no barrier and 4 represents complete barriers; +0, no facilitator and +4, complete facilitator.

The ICF core set qualifiers were obtained using sensitive and reliable instruments validated in Brazil (complete list of instruments included in Table S1). Responses (able or unable) on the Pediatric Evaluation of Disability Inventory (PEDI) were converted directly into ICF qualifiers, using a visual response card created by the researchers to facilitate parents’ or caregivers’ understanding when identifying the degree of difficulty experienced by the children in a category (Figure S2). The following instruments were applied to establish the remaining core set categories: Gross Motor Function Measure (GMFM–88), Visual Analog Scale (VAS), Infant Sleep Questionnaire (ISQ), and Modified Ashworth Scale and Goniometry. The GMFM scores, expressed in percentage, were directly converted into ICF qualifiers. The results of goniometry and the Modified Ashworth Scale were also directly converted into qualifiers. With the other instruments, the researchers used the visual response cards with the ICF qualifiers, so that the parents or caregivers could transform the responses given on the assessment instruments into qualifiers, based on the responses given on the assessment instruments.

For the categories that had no available tools, a specific questionnaire was applied to the parents or caregivers, whose responses were converted into ICF qualifiers during the interview, using visual response cards. The sociodemographic characteristics, head circumference, and other clinical data were collected from health records, physical examinations, imaging examination reports, and interviews with children and their parents. 

### 2.3. Translating Clinical Information and Standardized Assessment into the ICF Qualifiers

Some examples on how clinical information was translated into the ICF qualifiers is provided below:

- The category s110 (structure of brain) was determined considering the results of imaging exams (Nuclear Magnetic Resonance, Computed Tomography or Transfontanel Ultrasonography). For example, Computed Tomography demonstrating multiple calcifications at the cortical-white matter, predominating in temporal lobes, the qualifier 3 was assigned.
- The category b280 (sensation of pain) was evaluated using the Visual Analog Scale (VAS) by the question. When the answer was 9 or 10 at VAS, the qualifier 4 was assigned.
- The category d710 (Basic interpersonal interactions) was captured by the PEDI, using the Social function area, items F and G, interactive social game and interaction with friends, respectively. When the answer was 0, the qualifier 4 was assigned.
- The category e120 (Products and technology for personal indoor and outdoor mobility and transportation) was assessed by a self-developed question: “Does the child need assistive devices to help in locomotion? How much does this help or hinder the child’s functioning?” In order to translate this information into the ICF language, firstly, the caregiver stated if the assistive device was considered a facilitator or a barrier. Secondly, the caregiver’s perspective on how much this environmental factor influenced the child’s functioning was captured using the study visual response card (Figure S2). The caregiver’s response was mapped into the ICF qualifier (facilitator or barrier).

### 2.4. Ethical Principles

The study was approved by the Institutional Research Ethics Committee, under protocol number 2.357.552 and all the parents/caregivers gave their informed consent.

### 2.5. Statistical Analysis

The quantitative variables were expressed as mean ± standard deviation, and the categorical variables as percentages of the qualifiers, attributed in each category of the ICF core set.

## 3. Results

The general characteristics of the sample are shown in Table 1. Most of the children were girls, average age was 21 months and head circumference z-score ranged between 0.92 and −5.51. Around 65% of the parents lived with a minimum monthly wage (≈USD300.00), resulting from the benefit for their child.

As expected, technical images of the brain identified many structural abnormalities (s110). The following anatomical changes were predominant: punctiform calcifications, diffuse encephalic volume reduction, prominence of the supratentorial ventricular system, hypodensity of the white matter of the cerebral hemispheres, and accentuation of the grooves between the cerebellar fold.

Table 2 illustrates the percentages of disability for each ICF core set category, the function (b) and structure (s) of the body, and activity and participation (d). With respect to body function, the category most affected was b167 (mental functions of language), followed by severe disabilities, as demonstrated by the sample in categories b117 (intellectual functions), b710 (functions related to joint mobility), b735 (functions related to muscle tonus), and b760 (functions related to voluntary muscle control). However, no disability was found in participants for categories b134 (sleep functions) and b280 (pain sensation). In relation to body structure, 55.9% of the sample exhibited moderate disability in s110 (brain structure).

With respect to activity and participation level, 94.1% of participants were unable to perform age-appropriate fine motor hand movements (d440) and 70.6% to walk (d450); severe difficulty was observed in 55.9% of the sample in category d710 (basic personal interactions), but no difficulty for family relationships (d760).

Table 3 depicts each category of environmental factors identified as facilitator, barrier, or neither of the two. Complete facilitators considered for the sample were e310 (immediate family), e320 (friends), and e580 (health services, systems, and policies). The only complete barrier was category e460 (societal attitudes). Notably, 76.5% of the sample described no facilitator or barrier for e125 (communication products and technology).

**Figure 2:**
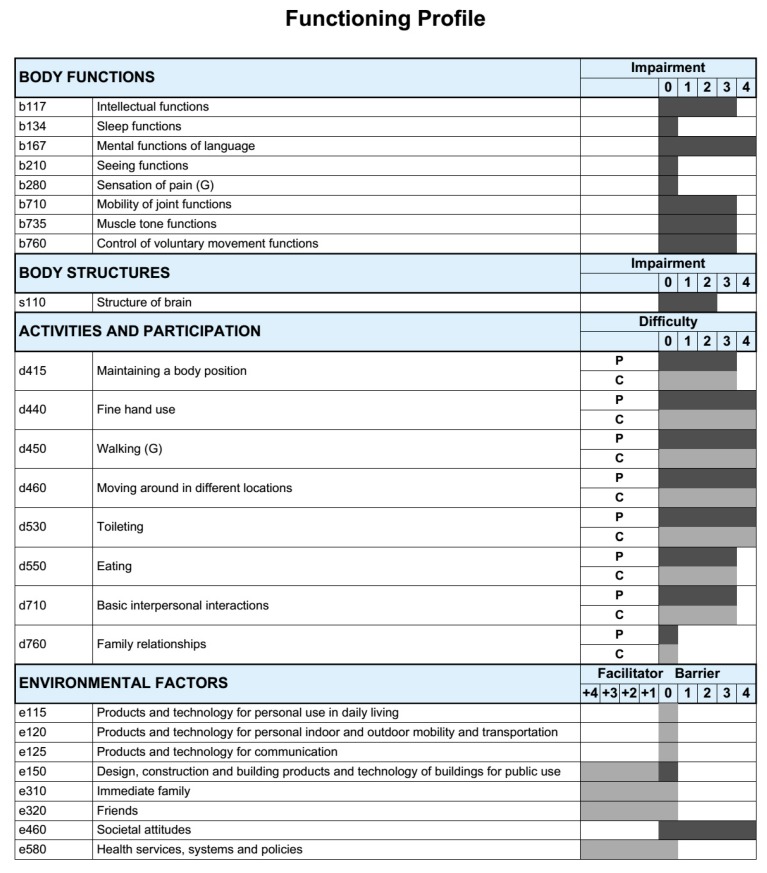
Functioning profile of the sample. The ICF qualifiers use to create the profile of functioning of this study sample represent the qualifiers that have the highest percentage within each ICF category. This profile of functioning was built using the ICF-based documentation form on this web page.

Using the ICF core set, a profile of functioning was built for the entire study sample (Figure 2). Within each category, the ICF qualifier with the highest percentage was used to create the profile. As shown in Figure 2, the profile of functioning provides a more detailed representation of the abilities and challenges of the children affected with ZIKV in our region. This profile of functioning was built using the ICF-based documentation form on this web page https://icf-core-sets.org/es/page0.php [18]. 

## 4. Discussion

This is the first Brazilian study to describe the functioning and disability profile of children with microcephaly caused by ZIKV, using tools based on the ICF. These results reveal the magnitude of disabilities or difficulties in function and body structure, activity and participation, as well as barriers and facilitators of the environment, which can direct therapeutic strategies and public policies for children with ZIKV-associated microcephaly. The data indicate that many functional areas showed complete disability, highlighting category b167 (mental functions of language), assessed by the Pediatric Evaluation of Disability Inventory (PEDI), the social function area, where 100% of the sample exhibited complete disability based on their age. This is an important finding as changes in language can compromise other functional areas, as demonstrated in a recent study, where children with communication and mobility problems experienced limitations in leisure activities, negatively affecting their long-term development, health, and well-being [19].

Nearly 95% of the sample demonstrated extreme difficulty in performing age-appropriate fine hand movements (d440), and they were unable to use any type of utensils or drinking vessels, indicating the need for a multidisciplinary approach and possible interventions with assistive technology [20]. The categories d450 (walking) and d530 (toileting) exhibited extreme difficulty in 70.6% and 47.1% of the sample, respectively. Assessment of gross motor function, conducted by the Gross Motor Function Measure (GMFM-88), identified severe disability in 64.7% of category d415 (maintaining body position). Likewise, Satterfield-Nash et al. (2017) [21] found severe motor impairment, assessed using the Hammersmith Infant Examination (HINE), in nearly 80% of the sample of children with microcephaly caused by ZIKV, including signs consistent with CP.

The repercussions of microcephaly associated with ZIKV in the domains activity and participation revealed severe difficulty in more than 70% of the sample in category d550 (eating). These results were expected since most of the sample were fed a diet of mashed, liquid, or strained food, findings similar to those observed in a follow-up of 19 children with ZIKV-related microcephaly where nearly half of the sample also exhibited eating disorders [21]. By contrast, around 90% of the sample was perceived by their parents as having no difficulty in the family relationships category (d760) and they lived harmoniously with their family. Although the families of children with disability may face challenges that are different from those of families with typically developed offspring. Similar to other studies [22,23], parents recognized the importance of having a participatory role in planning therapeutic services and reported that educating disabled children may have a positive impact on the family. 

The mobility of children with disabilities is commonly one of the main challenges for families and health professionals. In the present study, moving around (d460) was considered extremely difficult by nearly 70% of the sample, which can interfere significantly in a child’s interaction with an environment [24]. Indeed, research shows that the premature introduction of power wheelchairs to children up to 14 months of age improves psychosocial function and the ability to play [25]. In situations of scarce economic resources, such as those of the present study, where monthly income is approximately USD300.00, purchasing mobility aids such as a wheelchair may be unfeasible, and parents are obliged to wait long periods before receiving one from the National Health System, which is guaranteed by the National Plan for the Rights of People with Disability—Living without Limits Plan [26].

Interestingly, the present study found that 47.1% and 67.6% of participants reported no disability in the categories b134 (sleep functions) and b280 (pain sensation), respectively. Research demonstrates that children with disabilities who develop severe motor impairment are more susceptible to experiencing pain [27,28]. Although irritability was a symptom seen in the first clinical descriptions of children with ZIKV-related microcephaly in Brazil, none of the data were related to sleep pattern [16,29]. Of note, as this study was a one-time cross-sectional study, a prospective study with follow-up assessments might capture the impact of ZIKV on sleep and chronic pain in this population.

One of the main points of this study was using the ICF to identify the barriers and facilitators present in the environment, according to the perception of parents and caregivers of children with microcephaly caused by ZIKV, which may reflect the multiple influences of contextual factors on functioning and disability. While 85.3% of the sample reported category e310 (immediate family) as a complete facilitator, around 60% indicated category e320 (friends). These results reinforce the need for structured family and social support networks, since they are strong facilitators of social inclusion [30,31].

Access to services, systems, and policies can be converted into an important facilitator and indicator of positive functional results, especially in chronic conditions such as any childhood disability. In the present study, this point was assessed by category e580 (health services, systems, and policies), where more than 60% of the sample reported it as complete facilitator. These results may indicate that, despite the problems in regulating and accessing public health services faced by most of the Brazilian population, since microcephaly is a new condition, most of the needs of the children with this disorder enrolled in the study are being met. The Ministry of Health supports both basic and specialized care to provide better access to treatment and support for these children and their parents, respectively. The Brazilian National Health System offers basic care, specialized examinations and diagnoses, rehabilitation and hospital services, in addition to orthoses, prostheses, and walking aids [4]. 

It is also important to underscore the effort that most parents make to guarantee adherence to rehabilitation programs, reinforcing the importance of family-centered health care, which gives the families themselves the opportunity to establish their priorities and goals [32,33]. King and Chiarello (2014) [34] identified three essential factors for family-centered care: (1) respect for children and families, (2) recognition of the family’s impact on a child’s well-being, and (3) family–professional collaboration. The use of the ICF Core Sets could assist professionals to obtain a better understanding of the needs of the families and their children and therefore improve the family–professional collaboration. Understanding the parents’ perspective on therapeutic care is extremely important in enhancing the processes and results of the services [20].

Attitudinal barriers were assessed by category e460 (societal attitudes), where more than 40% of parents or caregivers report it as a complete barrier. This finding demonstrates the importance of awareness programs aimed at social inclusion and minimizing prejudice experienced by these families in the community, schools, or public services [35,36]. Studies have highlighted the importance of strengthening interventions in terms of context, since the environment will likely have more potential to change than domains such as body function and structure [37,38,39].

It is interesting to note that some aspects of the environment that are commonly relevant to children with disability, such as products and technologies for personal daily use (e115) and communication (e125), were reported by more than half the sample as being no barrier or facilitator, which may reflect a possible lack of information, an important aspect in the provision of care, of why and how to acquire products and technologies to benefit the functioning of their children [40]. Educating interested parties about the best ways of providing accessible services may minimize attitudinal barriers and facilitate the participation of children with disabilities [41].

The main limitation of the present study was the small sample size, because many of the children lived in rural areas with a poor public transport system, precluding coming to rehabilitation centers during data collection. We plan to assess these families during home visits in the future. Another disadvantage was in relation to the homogeneity of the sample, composed of children of very low economic level.

Given its relevance, assessments based on the ICF are important to plan comprehensive assessment, also in educating professionals about the needs of patients and their families, as well as intervention priorities [14,42,43]. Implementing ICF-based changes in clinical practice requires organizational planning by all the professionals of a multidisciplinary team [44]. As such, the use of the Common Brief ICF core set for CP enabled a detailed description of functioning and disability in children with ZIKV-associated microcephaly from four rehabilitation centers. The study also made it possible to train teams in the use of ICF and identify the needs and intervention goals for this group of children that will guide the rehabilitation program, with a focus on the family and child’s functioning.

## 5. Conclusions

Our findings reinforce the need to maximize health care and make access to universal information, based on the ICF, for multiprofessional teams, administrators, family members, and children. Health care for the ZIKV affected population should be family-centered, considering modifiable environmental factors and highlighting the functional objectives, in order to ensure optimal levels of participation in domestic, school, and community activities in our region. Additional research is needed in order to expand the ICF-focused evaluation model for children with ZIKV-associated microcephaly to other regions of Brazil, as well as to investigate the intervention strategies that are being targeted to the target audience.

## Figures and Tables

**Table 1:** Sociodemographic and clinical data. ZIKV: Zika virus.

**Characteristics of the Children (*n* = 34)**	
Sex	Male = 47.0% Female = 52.9%
Age (months)	Mean = 21.2 ± 7.3
Height (cm)	43.5 ± 8.3
Weight (grams)	2773.1 ± 462.4
1′Apgar	8.2 ± 1.0
5′Apgar	9.0 ± 0.5
Score-Z (Head circumference)	0.92 to −5.51
Use of ventilator support	Yes = 23.5% No = 76.5%
Exclusive breastfeeding	Yes = 47.1% No = 52.9%
Owns baby carriage or wheelchair	Yes = 55.9% No = 44.1%
**Characteristics of the Mothers (*n* = 34)**	
Age (years)	27.0 ± 7.0
Location of residence	100% urban zone
Schooling	Complete elementary education = 17.6% Incomplete elementary education = 14.7% Complete secondary education = 47.1% Incomplete secondary education = 8.8% Incomplete university education = 11.8%
Income	One minimum monthly salary = 64.7% Two to three minimum monthly salaries= 35.3%
Number of pregnancies	1 = 44.1% 2 = 35.3% 3 = 14.7% 4 = 5.9%
Gestational age (weeks)	37.7 ± 2.6
Type of delivery	Normal = 41.2% Cesarean = 58.8%
Number of prenatal visits	7.7 ± 2.9
Number of ultrasounds	4.8 ± 2.2
Threatened miscarriage	Yes = 23.5% No = 76.5%
Symptoms of ZIKV	Yes = 79.4% No = 20.6%
Trimester of pregnancy when ZIKV symptoms emerged	None = 20.6% 1st = 47.1% 2nd = 17.6% 3rd = 14.7%
Diagnosis of Zika virus infection during pregnancy	Yes = 67.6% No = 32.4%

**Table 2:** Relative frequency of children with microcephaly by ZIKV in body functions, body structures, and activities/participation categories of the Brief Common ICF (International Classification of Functioning Disability and Health) Core Set for cerebral palsy (CP) (*n* = 34).

**Category Functions**		**Qualifier 0** **None Problem**	**Qualifier 1** **Mild Problem**	**Qualifier 2** **Moderate Problem**	**Qualifier 3** **Severe Problem**	**Qualifier 4** **Complete Problem**	**Qualifier 9** **Not Applicable**
b117	Intellectual functions	2.9	8.8	-	55.9	32.4	-
b134	Sleep functions	47.1	11.8	11.8	8.8	20.6	-
b167	Mental functions of language	-	-	-	-	100.0	-
b210	Seeing functions	32.4	14.7	17.6	29.4	5.9	-
b280	Sensation of pain	67.6	2.9	5.9	11.8	11.8	-
b710	Mobility of joint functions	-	8.8	14.7	64.7	11.8	-
b735	Muscle tone functions	-	5.9	5.9	76.5	11.8	-
b760	Control of voluntary movement functions	-	5.9	5.9	70.6	17.6	-
**Category**	**Body Structures**	**Qualifier 0** **None Problem**	**Qualifier 1** **Mild Problem**	**Qualifier 2** **Moderate Problem**	**Qualifier 3** **Severe Problem**	**Qualifier 4** **Complete Problem**	**Qualifier 9** **Not Applicable**
s110	Structure of brain	-	-	55.9	44.1	-	-
**Category**	**Activities/Participation**	**Qualifier 0** **None Problem**	**Qualifier 1** **Mild Problem**	**Qualifier 2** **Moderate Problem**	**Qualifier 3** **Severe Problem**	**Qualifier 4** **Complete Problem**	**Qualifier 9** **Not Applicable**
d415	Maintaining a body position	-	8.8	8.8	64.7	17.6	-
d440	Fine hand use	-	-	-	5.9	94.1	-
d450	Walking	-	-	-	-	70.6	29.4
d460	Moving around in different locations	2.9	-	11.8	17.6	67.6	-
d530	Toileting	-	-	-	23.5	47.1	29.4
d550	Eating	11.8	2.9	2.9	70.6	11.8	-
d710	Basic interpersonal interactions	-	-	2.9	55.9	41.2	-
d760	Family relationships	88.2	2.9	2.9	2.9	2.9	-

**Table 3:** Relative frequency of children with microcephaly by ZIKV in environmental factors categories of the Brief Common ICF Core Set for CP (*n* = 34).

Category		Mild Barrier 1	Moderate Barrier 2	Severe Barrier 3	Complete Barrier 4	No Facilitator/No Barrier 0	Mild Facilitator +1	Moderate Facilitator +2	Substancial Facilitator +3	Complete Facilitator +4
e115	Products and technology for personal use in daily living	5.9	-	2.9	2.9	50.0	5.9	2.9	17.6	11.8
e120	Products and technology for personal indoor and outdoor mobility and transportation	2.9	20.6	-	-	35.3	-	-	8.8	32.4
e125	Products and technology for communication	-	-	-	8.8	76.5	-	-	-	14.7
e150	Design, construction and building products and technology of buildings for public use	-	-	5.9	14.7	32.4	2.9	2.9	8.8	32.4
e310	Immediate Family	-	-	-	-	5.9	-	-	5.9	85.3
e320	Friends	-	-	-	-	20.6	-	-	20.6	58.8
e460	Societal attitudes	-	2.9	20.6	41.2	35.3	-	-	-	-
e580	Health services, systems and policies	-	-	-	17.6	-	-	-	20.6	61.8

## Supplementary Materials

The following are available online at http://www.mdpi.com/1660-4601/15/6/1107/s1, Figure S1: Brief Common ICF Core Set for CP, Figure S2: Visual response cards. (a) Visual response card for body function categories, (b) Visual response card for activities and participation categories, (c) Visual response card for environmental factors categories. Table S1: Tools used to apply the Brief Common ICF Core Set for CP. GMFM: Gross Motor Function Measure.
