# Supplementary material for: Functioning and Disability Profile of Children with Microcephaly Associated with Congenital Zika Virus Infection
Source: Int J Environ Res Public Health. 2018 May 29;15(6):1107. doi: 10.3390/ijerph15061107 (PMC6025082; doi:10.3390/ijerph15061107)
Supplement: Supplementary file 1 [file ijerph-15-01107-s001.zip › ijerph-299640-SI.pdf]

International Journal of
Environmental Research and Public Health

MDPI

BRIEF COMMON ICF CORE SET FOR CHILDREN & YOUTH WITH CEREBRAL PALSY

From 0 to 18 years

N=25 ICF Categories

**BODY STRUCTURES (n=1)**<br>= anatomical parts of the body such as organs, limbs and their components	
**s110**	**Structure of brain**

**BODY FUNCTIONS (n=8)**<br>= physiological functions of body systems (including psychological functions)	
**b117**	**Intellectual functions**
	**General mental functions, required to understand and constructively integrate the various mental functions, including all cognitive functions and their development over the life span.**<br>*Inclusions: functions of intellectual growth; intellectual retardation, mental retardation, dementia*<br>*Exclusions: memory functions (b144); thought functions (b160); basic cognitive functions (b163); higher-level cognitive functions (b164)*
**b134**	**Sleep functions**
	**General mental functions of periodic, reversible and selective physical and mental disengagement from one's immediate environment accompanied by characteristic physiological changes.**<br>*Inclusions: functions of amount of sleeping, and onset, maintenance and quality of sleep; functions involving the sleep cycle, such as in insomnia, hypersomnia and narcolepsy*<br>*Exclusions: consciousness functions (b110); energy and drive functions (b130); attention functions (b140); psychomotor functions (b147)*
**b167**	**Mental functions of language**
	**Specific mental functions of recognizing and using signs, symbols and other components of a language.**<br>*Inclusions: functions of reception and decryption of spoken, written or other forms of language such as sign language; functions of expression of spoken, written or other forms of language; integrative language functions, spoken and written, such as involved in receptive, expressive, Broca's, Wernicke's and conduction aphasia*<br>*Exclusions: attention functions (b140); memory functions (b144); perceptual functions (b156); thought functions (b160); higher-level cognitive functions (b164); calculation functions (b172); mental functions of complex movements (b176); Chapter 2 Sensory Functions and Pain; Chapter 3 Voice and Speech Functions*
**b210**	**Seeing functions**
	**Sensory functions relating to sensing the presence of light and sensing the form, size, shape and colour of the visual stimuli.**<br>*Inclusions: visual acuity functions; visual field functions; quality of vision; functions of sensing light and colour, visual acuity of distant and near vision, monocular and binocular vision; visual picture quality; impairments such as myopia, hypermetropia, astigmatism, hemianopia, colour-blindness, tunnel vision, central and peripheral scotoma, diplopia, night blindness and impaired adaptability to light*<br>*Exclusion: perceptual functions (b156)*
**b280**	**Sensation of pain**
	**Sensation of unpleasant feeling indicating potential or actual damage to some body structure.**<br>*Inclusions: sensations of generalized or localized pain in one or more body part, pain in a dermatome, stabbing pain, burning pain, dull pain, aching pain; impairments such as myalgia, analgesia and hyperalgesia*
**b710**	**Mobility of joint functions**
	**Functions of the range and ease of movement of a joint.**<br>*Inclusions: functions of mobility of single or several joints, vertebral, shoulder, elbow, wrist, hip, knee, ankle, small joints of hands and feet; mobility of joints generalized; impairments such as in hypermobility of joints, frozen joints, frozen shoulder, arthritis Exclusions: stability of joint functions (b715); control of voluntary movement functions (b760)*
**b735**	**Muscle tone functions**
	**Functions related to the tension present in the resting muscles and the resistance offered when trying to move the muscles passively.**<br>*Inclusions: functions associated with the tension of isolated muscles and muscle groups,muscles of one limb, one side of the body and the lower half of the body, muscles of all limbs, muscles of the trunk, and all muscles of the body; impairments such as hypotonia, hypertonia and muscle spasticity, myotonia and paramyotonia*<br>*Exclusions: muscle power functions (b730); muscle endurance functions (b740)*
**b760**	**Control of voluntary movement functions**
	Functions associated with control over and coordination of voluntary movements.<br>*Inclusions: functions of control of simple voluntary movements and of complex voluntary movements, coordination of voluntary movements, supportive functions of arm or leg, right left motor coordination, eye hand coordination, eye foot coordination; impairments such as control and coordination problems, e.g. clumsiness and dysdiadochokinesia*<br>*Exclusions: muscle power functions (b730); involuntary movement functions (b765); gait pattern functions (b770)*

.

**ACTIVITIES AND PARTICIPATION (n=8)**<br>= execution of a task or action by an individual and involvement in a life situation	
**d415**	**Maintaining a body position**
	**Staying in the same body position as required, such as remaining seated or remaining standing for work or school.**<br>*Inclusions: maintaining a lying, squatting, kneeling, sitting and standing position*
**d440**	**Fine hand use**
	**Performing the coordinated actions of handling objects, picking up, manipulating and releasing them using one's hand, fingers and thumb, such as required to lift coins off a table or turn a dial or knob.**<br>*Inclusions: picking up, grasping, manipulating and releasing*
**d450**	**Walking**
	**Moving along a surface on foot, step by step, so that one foot is always on the ground, such as when strolling, sauntering, walking forwards, backwards or sideways.**<br>*Inclusions: walking short or long distances; walking on different surfaces; walking around obstacles Exclusions: transferring oneself (d420); moving around (d455)*<br>*Exclusions: transferring oneself (d420); moving around (d455)*
**d460**	**Moving around in different locations**
	**Walking and moving around in various places and situations, such as walking between rooms in a house, within a building, or down the street of a town..**<br>*Inclusions: moving around within the home, crawling or climbing within the home; walking or moving within buildings other than the home, and outside the home an dother buildings*
**d530**	**Toileting**
	**Indicating the need for, planning and carrying out the elimination of human waste (menstruation, urination and defecation), and cleaning oneself afterwards.**<br>*Inclusions: regulating urination, defecation and menstrual care*<br>*Exclusions: washing oneself (d510); caring for body parts (d520)*
**d550**	**Eating**
	**Indicating need for, and carrying out the coordinated tasks and actions of eating food that has been served, bringing it to the mouth and consuming it in culturally acceptable ways, cutting or breaking food into pieces, opening bottles and cans, using eating implements, having meals, feasting or dining.**<br>*Exclusion: drinking (d560)*
**d710**	**Basic interpersonal interactions**
	**Interacting with people in a contextually and socially appropriate manner, such as by showing consideration and esteem when appropriate, or responding to the feelings of others.**<br>*Inclusions: showing respect, warmth, appreciation, and tolerance in relationships; responding to criticism and social cues in relationships; and using appropriate physical contact in relationships*
**d760**	**Family relationships**
	**Creating and maintaining kinship relationships, such as with members of the nuclear family, extended family, foster and adopted family and step-relationships, more distant relationships such as second cousins or legal guardians.**<br>*Inclusions: parent-child and child-parent relationships, sibling and extended family relationships*

**ENVIRONMENTAL FACTORS (n=8)**<br>= make up the physical, social and attitudinal environment in which people live and conduct their lives	
**e115**	**Products and technology for personal use in daily living**
	**Equipment, products and technologies used by people in daily activities, including those adapted or specially designed, located in, on or near the person using them.**<br>*Inclusions: general and assistive products and technology for personal use*<br>*Exclusions: products and technology for personal indoor and outdoor mobility and transportation (e120); products and technology for communication (e125)*
**e120**	**Products and technology for personal indoor and outdoor mobility and transportation**
	**Equipment, products and technologies used by people in activities of moving inside and outside buildings, including those adapted or specially designed, located in, on or near the person using them.**<br>*Inclusions: general and assistive products and technology for personal indoor and outdoor mobility and transportation*
**e125**	**Products and technology for communication**
	**Equipment, products and technologies used by people in activities of sending and receiving information, including those adapted or specially designed, located in, on or near the person using them.**<br>*Inclusions: general and assistive products and technology for communication*
**e150**	**Design, construction and building products and technology of buildings for public use**
	**Products and technology that constitute an individual's indoor and outdoor human-made environment that is planned, designed and constructed for public use, including those adapted or specially designed.**<br>*Inclusions: design, construction and building products and technology of entrances and exits, facilities and routing*
**e310**	**Immediate family**
	**Individuals related by birth, marriage or other relationship recognized by the culture as immediate family, such as spouses, partners, parents, siblings, children, foster parents, adoptive parents and grandparents.**<br>*Exclusions: extended family (e315); personal care providers and personal assistants (e340)*

.

**ENVIRONMENTAL FACTORS (n=8)**<br>= make up the physical, social and attitudinal environment in which people live and conduct their lives	
**e320**	**Friends**
	**Individuals who are close and ongoing participants in relationships characterized by trust and mutual support.**
**e460**	**Societal attitudes**
	**General or specific opinions and beliefs generally held by people of a culture, society, subcultural or other social group about other individuals or about other social, political and economic issues that influence group or individual behaviour and actions.**
**e580**	**Health services, systems and policies**
	**Services, systems and policies for preventing and treating health problems, providing medical rehabilitation and promoting a healthy lifestyle.**<br>*Exclusion: general social support services, systems and policies (e575)*

Figure S1. Brief Common ICF Core Set for CP.

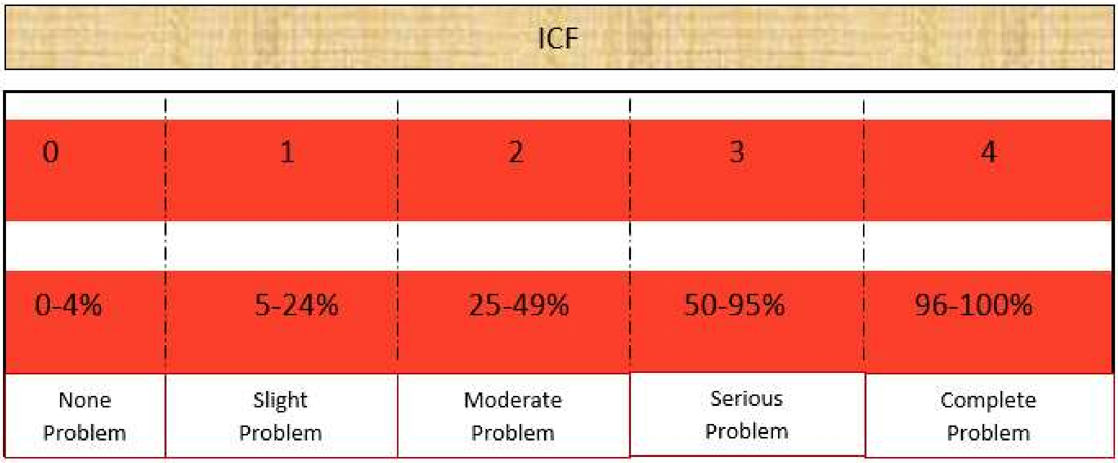
ICF
0
1
2
3
4
0-4%
5-24%
25-49%
50-95%
96-100%
None Problem
Slight Problem
Moderate Problem
Serious Problem
Complete Problem

(a)

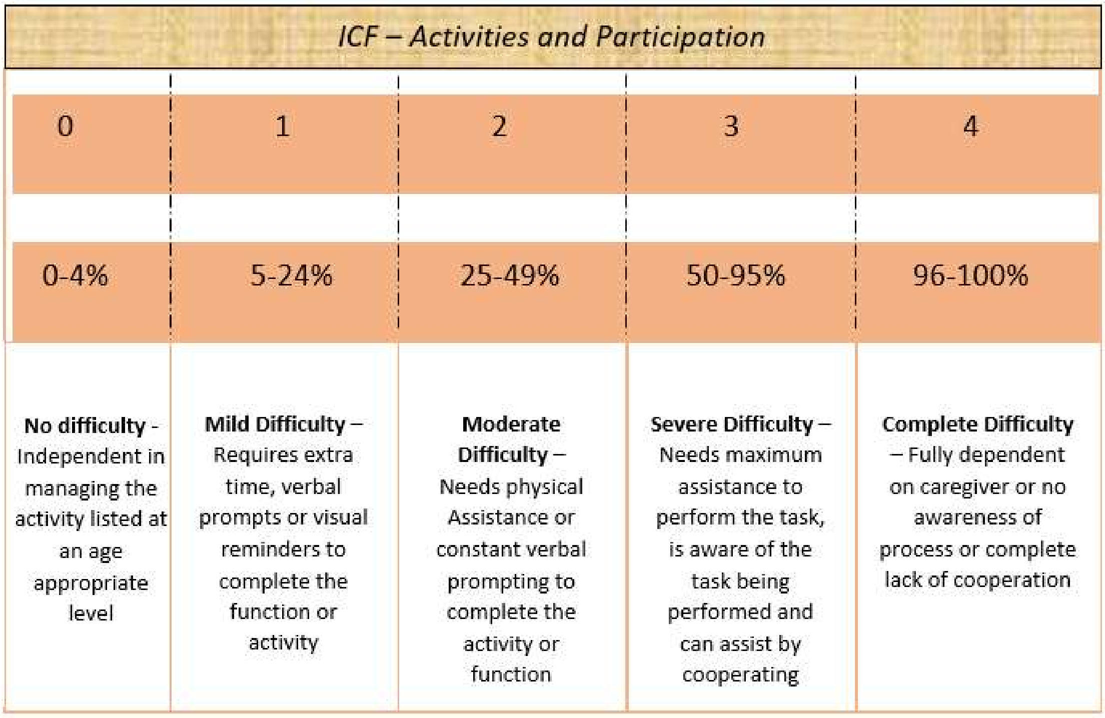
ICF – Activities and Participation
0
1
2
3
4
0-4%
5-24%
25-49%
50-95%
96-100%
No difficulty - Independent in managing the activity listed at an age appropriate level
Mild Difficulty – Requires extra time, verbal prompts or visual reminders to complete the function or activity
Moderate Difficulty – Needs physical Assistance or constant verbal prompting to complete the activity or function
Severe Difficulty – Needs maximum assistance to perform the task, is aware of the task being performed and can assist by cooperating
Complete Difficulty – Fully dependent on caregiver or no awareness of process or complete lack of cooperation

(**b**)

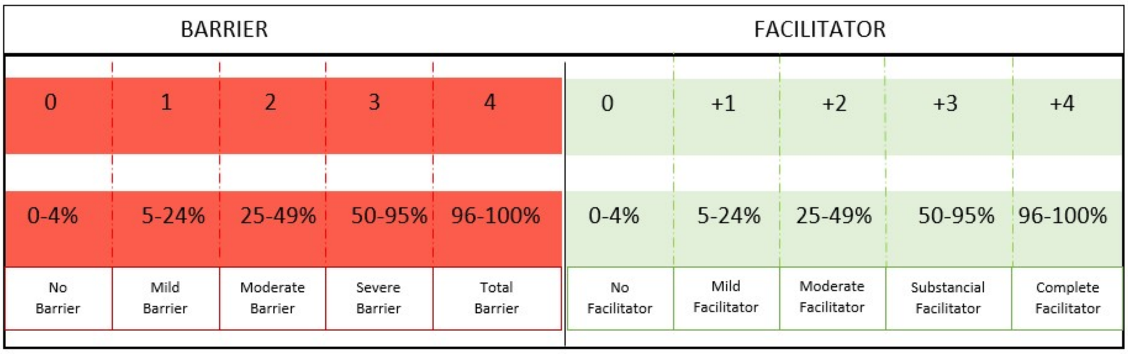
BARRIER
FACILITATOR
0
1
2
3
4
0
+1
+2
+3
+4
0-4%
5-24%
25-49%
50-95%
96-100%
0-4%
5-24%
25-49%
50-95%
96-100%
No Barrier
Mild Barrier
Moderate Barrier
Severe Barrier
Total Barrier
No Facilitator
Mild Facilitator
Moderate Facilitator
Substancial Facilitator
Complete Facilitator

(c)

Figure S2. Visual response cards. (**a**) Visual response card for body function categories, (**b**) Visual response card for activities and participation categories**,** (**c**) Visual response card for environmental factors categories.

Table S1. Tools used to apply the Brief Common ICF Core Set for CP. ~~GMFM: Gross Motor Function Measure.~~

ICF	Descriptor	Tools	
b117	Intellectual functions	Pediatric Evaluation of Disability Inventory (PEDI)	Social function area, items A and B, understanding the meaning of words and complex sentences, respectively
b134	Sleep functions	Infant Sleep Questionnaire (ISQ)	Assesses the perception of parents regarding the child's sleeping behavior
b167	Mental functions of language	PEDI	Social function area, items C and D: Functional use of communication and complexity of expressive communication, respectively
b210	Seeing functions	Does the child have problems seeing? Has the child had an eye examination?	Questionnaire applied to the mother in the form of an interview
b280	Sensation of pain	Is the child experiencing pain in any part of the body? Visual Analog Scale (VAS)	Questionnaire applied to the mother in the form of an interview
b710	Mobility of joint functions	Goniometry	Of upper and lower limbs in order to identify possible contractions, movement limitations and joint deformities
b735	Muscle tone functions	Modified Ashworth Scale	Applied manually by the therapist to determine muscle resistance for passive stretching, providing a qualitative measure
b760	Control of voluntary movement functions	Gross Motor Function Measure (GMFM-88)	Domains A—lying down and rolling and B - sitting
d415	Maintaining a body position	GMFM-88	Domains A—lying down and rolling and B - sitting
d440	Fine hand use	PEDI	Self-care area, items B and C, using utensils and drinking receptacles, respectively
d450	Walking	GMFM-88	Domain E—walking, running and jumping
d460	Moving around in different locations	How does the child move around inside and outside the home?	Questionnaire applied to the mother in the form of an interview.
d530	Toileting	PEDI	Self-care area, items N and O, urinary and intestinal control, respectively
d550	Eating	PEDI	Self-care area, item A, food texture

d710	Basic interpersonal interactions	PEDI	Social function area, items F and G, interactive social game and interaction with friends, respectively
d760	Family relationships	Who lives with the child? How is your relationship with the child? With the other family members?	Questionnaire applied to the mother in the form of an interview.
e115	Products and technology for personal use in daily living	How does equipment for ADL facilitate or hinder the child's life?	Questionnaire applied to the mother in the form of an interview.
e120	Products and technology for personal indoor and outdoor mobility and transportation	Does the child need assistive devices to help in locomotion? How much does this help or hinder the child's life?	Questionnaire applied to the mother in the form of an interview.
e125	Products and technology for communication	How do communication devices facilitate or hinder the child's life?	Questionnaire applied to the mother in the form of an interview.
e150	Design, construction and building products and technology of buildings for public use	Does the child need help to enter or move around public places, such as ramps? Are these adaptations present in health centers and rehabilitation facilities that the child attends?	Questionnaire applied to the mother in the form of an interview.
e310	Immediate family	Does the child receive physical or emotional support from parents and siblings? How does this affect their functioning?	Questionnaire applied to the mother in the form of an interview.
e320	Friends	Does the child interact with other children? Do these children treat your child well?	Questionnaire applied to the mother in the form of an interview.
e460	Societal attitudes	Does the child suffer discrimination? Can you describe one of these experiences?	Questionnaire applied to the mother in the form of an interview.
e580	Health services, systems and policies	Is the child undergoing rehabilitation? Has the child undergone or waiting to undergo examinations or surgery?	Questionnaire applied to the mother in the form of an interview.
s110	Structure of brain	Imaging examination results	Assessed based on imaging examination results - cranial computerized tomography cranioencephalic magnetic resonance, transfontanellar ultrasound and cranial radiography

International Journal of
*Environmental Research
and Public Health*
